# Wernicke's Encephalopathy Complicating Hyperemesis during Pregnancy

**DOI:** 10.1155/2016/8783932

**Published:** 2016-02-16

**Authors:** Mohamed Adnane Berdai, Smael Labib, Mustapha Harandou

**Affiliations:** Department of Obstetric and Pediatric Anaesthesiology and Intensive Care, University Hospital Hassan II, 30070 Fez, Morocco

## Abstract

Wernicke's encephalopathy is caused by severe thiamine deficiency; it is mostly observed in alcoholic patients. We report the case of a 28-year-old woman, at 17 weeks of gestational age, with severe hyperemesis gravidarum. She presented with disturbance of consciousness, nystagmus, ophthalmoplegia, and ataxia. The resonance magnetic imagery showed bilaterally symmetrical hyperintensities of thalamus and periaqueductal area. The case was managed with very large doses of thiamine. The diagnosis of Wernicke's encephalopathy was confirmed later by a low thiamine serum level. The patient was discharged home on day 46 with mild ataxia and persistent nystagmus. Wernicke's encephalopathy is a rare complication of hyperemesis gravidarum. It should be diagnosed as early as possible to prevent long-term neurological sequela or death. Thiamine supplementation in pregnant women with prolonged vomiting should be initiated, especially before parenteral dextrose infusion. Early thiamine replacement will reduce maternal morbidity and fetal loss rate.

## 1. Introduction

Wernicke's encephalopathy (WE) is a central neurological disorder, characterised by the classic triad of encephalopathy, ophthalmoplegia, and/or nystagmus and ataxia [1]. It is resulting from thiamine deficiency and is associated with significant morbidity and mortality [2]. WE is mostly seen in alcoholics but can also occur in any malnourished state. We report an unusual etiology of WE: hyperemesis gravidarum (HG).

## 2. Case Report

A 28-year-old woman, G2P1, at 17 weeks of gestation, with unremarkable medical history, started vomiting during the 6th week of gestation for which she was treated with antiemetics, but she continued to have persistent vomiting and complained of general weakness. At 16 weeks of gestation, she complained of diplopia and progressively presented deterioration of consciousness. At this stage, she had lost over 10% of her body weight. She was treated with intravenous fluids including dextrose and metoclopramide and was referred to our tertiary care institution.

At admission, we found a drowsy patient with a Glasgow Coma Scale (GCS) of 13/15. She was not oriented to place and time. The neurological examination revealed loss of equilibrium with incoordination of gait and trunk ataxia. The pupils were bilaterally equal and reactive and ocular fundus was normal. Ocular movements showed restricted bilateral convergence, left lateral rectus palsy with multidirectional nystagmus.

**Figure 1:**
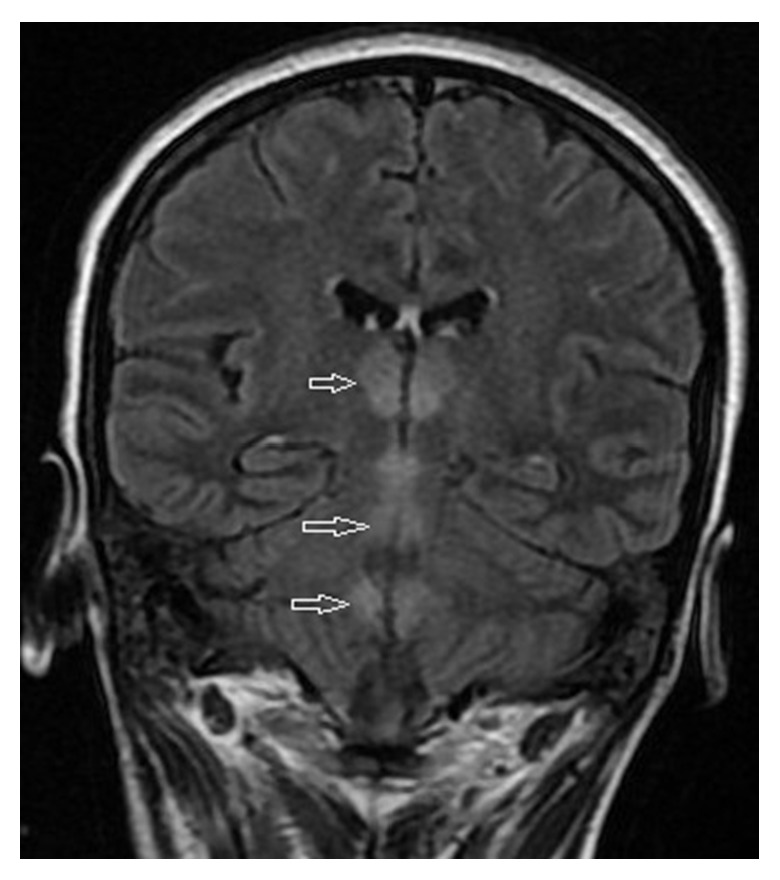
Magnetic resonance imaging of brain showed symmetrical hypersignal intensity at bilateral posterior thalamus and periaqueductal area on fluid-attenuated inversion recovery series.

The blood pressure was 120/90 mmHg, pulse was 138/min, and respiratory rate was 16/min; no edema was present and the patient was afebrile. Kidney function tests showed a kidney injury with serum creatinine level at 274 *μ*mol/L (normal range (NR): 50–100 *μ*mol/L) and urea at 20.8 mmol/L (NR: 2.5–6.1 mmol/L). Liver function tests showed L-aspartate aminotransferase level at 157 IU/L (NR < 40) and L-alanine aminotransferase at 113 IU/L (NR < 45). The serum potassium level was 2.4 mEq/L. Due to deterioration of consciousness (GCS at 8), the patient was intubated and artificially ventilated. Cerebrospinal fluid (CSF) examination and cranial magnetic resonance imaging (MRI) were also undertaken to exclude other intracranial causes. The CSF findings were normal, but the MRI revealed the following in T2 sequences, in fluid-attenuated inversion recovery (FLAIR), and in diffusion weighted imaging (DWI): bilaterally symmetrical hyperintensities in medial and posterior thalamic and in periaqueductal area (Figure 1). Venous and arterial magnetic resonance angiography was normal.

With nystagmus, ophthalmoplegia, ataxia, and confusion in a patient with inadequate intake of thiamine due to HG, and also specific cerebral images, a provisional diagnosis of WE was made in this case. As a serum thiamine level was not available immediately, she was started on parenteral thiamine 500 mgs twice daily and normal saline infusion. She received also potassium supplementation. Fetal heart sound was absent and antenatal ultrasonogram showed single nonvital fetus of 17 weeks of gestation. Medical termination of pregnancy was conducted. Thiamine serum level performed at admission in another institution was inferior to 36 nmol/L (NR: 67–200 nmol/L), confirming the diagnosis of WE. Despite the neurological improvement, the patient could not be weaned off ventilatory support and tracheotomy was then performed. By day 35, the patient was conscious; the neurological examination revealed tetraparesis due to long stay in intensive care unit and ataxia. Later, she tolerated oral feeds, shifted to oral thiamine, and was able to walk. She was discharged home on day 46 with mild ataxia and horizontal end gaze nystagmus. At this time, she had normal cognitive function and preserved memory.

## 3. Discussion

WE is mostly seen in alcoholics but can also occur in any malnourished state. The prevalence of WE in a nonalcoholic patient varies from 0.04% to 0.13% [3]. It should be considered in patients with anorexia nervosa, prolonged vomiting associated with chemotherapy, gastrointestinal disease, haemodialysis, and HG [4]. In a large literature review, the most frequent causes of WE in nonalcoholic patients were neoplastic disease (18.1%) and gastrointestinal surgery (16.8%) [5].

Thiamine pyrophosphate is the biological active form of vitamin B1; it is an essential coenzyme in many biochemical pathways in the brain, including transketolase, alpha-ketoglutarate dehydrogenase, and pyruvate dehydrogenase [2]. The mechanism through which its deficit causes brain lesions is unknown, although it is believed that neuronal damage begins once the metabolism in brain regions with high metabolic requirements and high thiamine turnover is inhibited [1]. Time to deplete the body's store of thiamine is about 3 weeks. The daily requirement of thiamine is around 1.1 mg/day for females, and it increases to 1.5 mg/day, particularly during pregnancy and lactation [6], and even more by the impaired absorption due to HG.

Although WE is reversible, major complications can arise in the pregnant woman and her unborn child. On the maternal side, without active management, WE can lead to permanent neurologic lesions and Korsakoff syndrome, which is fatal in 10–20% of cases. On the fetal side, WE can lead to miscarriage, preterm birth, and intrauterine growth retardation [3]. The typical lesions in MRI are symmetrical and located in the thalamus, mammillary bodies, tectal plate, and periaqueductal area; early reversible cytotoxic oedema is the most distinctive lesion [1]. The diagnosis of WE is based on the clinical manifestations and rapid reversal of symptoms with thiamine. Determination of blood transketolase activity and thiamine pyrophosphate reflects the thiamine status in the body [7].

Our patient presented with the classical clinical triad following intractable vomiting and dextrose administration without thiamine supplementation. MRI also detected bilateral and symmetric specific lesions. The clinical and radiological presentation was in favor of high suspicion of an acute thiamine deficiency, indicating immediate thiamine supplementation.

There are no universally accepted guidelines regarding the optimal dose, best route, and time of administration of thiamine. The European Federation of Neurological Societies recommends that thiamine should be given at a dose of 200 mg thrice daily via intravenous route, started before any carbohydrate, and continued until there is no further improvement in signs and symptoms [5]. Due to clinical severity of our case, we opted for a high thiamine dose of 500 mg twice daily, which led later to an important clinical improvement.

However, the complete remission of WE in pregnant women is rarely obtained, possibly related to insufficient thiamine supplementation. Chiossi et al. [6] in a review of 49 reported cases concluded that symptoms resolution required often months and complete remission is obtained in only 14 cases, spontaneous fetal loss rate was 37%, and elective abortion rate was 10% [6].

In conclusion, the delay in appropriate management of our case was responsible for intrauterine fetal death and persistent ataxia. Therefore, any pregnant woman with frequent vomiting should receive thiamine supplements, and if she develops any neurological manifestation, WE should be considered.
